# Curdlan Incorporation Enhances the Cooking, Rheological, and Textural Attributes of Thermally Sterilized Rice Noodles

**DOI:** 10.3390/foods14040674

**Published:** 2025-02-17

**Authors:** Jing Wang, Yongxin Liu, Qingjie Sun, Man Li, Yanfei Wang, Fengwei Xie

**Affiliations:** 1 Ministry of Agriculture and Rural Affairs, Key Laboratory of Agro-Products Processing, Institute of Food Science and Technology, Chinese Academy of Agricultural Sciences, Beijing 100193, China; wj17862875696@126.com; 2 College of Food Science and Engineering, Qingdao Agricultural University, Qingdao 266109, China; yongxin1130@163.com (Y.L.); phdsun@163.com (Q.S.); 3 Nottingham Ningbo China Beacons of Excellence Research and Innovation Institute, University of Nottingham Ningbo China, 211 Xingguang Road, Ningbo 315048, China; fwhsieh@gmail.com

**Keywords:** thermal sterilization, curdlan, rice noodles, high-moisture starch food, polysaccharide gels, rice noodle texture

## Abstract

Thermal treatment of rice starch, which is the main ingredient in rice noodles and has cooling-set gelling behavior, can disrupt hydrogen bonding, leading to a compromised gel structure. This can lead to a softer texture and reduced textural attributes and cooking characteristics of rice noodles. This study investigated how thermal sterilization and curdlan integration affect the rheological characteristics, microstructure, and quality of rice noodles. Fourier-transform infrared (FTIR) spectroscopy, kinetic analysis, and scanning electron microscopy (SEM) confirmed that the incorporation of curdlan, a thermally set polysaccharide gel, enhances hydrogen bonding, accelerates gel formation, and yields a denser gel structure to rice noodles. This enhancement improves solid-like behavior, storage modulus, textural properties, and cooking characteristics. Compared to pure rice noodles subjected to thermal sterilization, rice noodles incorporating 2.0% curdlan showed reductions of 74.71% in cooking breakage rate and 68.18% in cooking loss rate. Conversely, hardness and springiness increased by 19.82% and 18.75%, respectively. This study offers valuable insights for developing high-quality fresh rice noodles.

## 1. Introduction

Rice noodles, a staple of Chinese cuisine with a history spanning over 2000 years, hold a significant market presence in China and Southeast Asia. According to the difference in water content, rice noodles are categorized into dried, semi-dried, and fresh rice noodles [1]. Presently, fresh rice noodles are attracting growing attention for their high elasticity, enticing taste, distinct flavor, and tender texture [2].

Fresh rice noodles, once thermally sterilized, have a shelf life of over six months. They are prepared by boiling in water for 2–3 minutes before consumption, which makes them highly convenient for consumers. However, thermal treatment could weaken the hydrogen-bonded network structure of starch in rice noodles. This can lead to quality deterioration, particularly noticeable in the form of broken strips and a fragile quality [3]. Previous research has extensively explored the quality enhancement of rice noodles, focusing on the effect of various food additives or natural colloids on the quality attributes of products without heating treatment [4,5]. Yet, there is limited research addressing quality enhancement post-thermal sterilization of rice noodles.

Curdlan (CD) is a food additive approved by the FDA and used as a multifunctional thickener and stabilizer in food [6]. As a microbial polysaccharide, CD has distinctive gelling characteristics, particularly the capacity to generate a thermally irreversible gel above 80 °C [7,8]. The gel structure can further densify upon repeated heating, attracting considerable attention in recent years [9]. CD has demonstrated positive effects on starch-based food quality. It is reported that the addition of 0.6% CD could improve the hardness of dough with a high storage modulus [10]. Meanwhile, CD can also elevate the cooking and textural properties of noodles by inhibiting amylose leaching during gelatinization [11]. CD plays a crucial role in enhancing the rehydration and texture properties of dry rice noodles due to its excellent water retention properties [12]. Furthermore, a previous study on the effect of CD on fresh rice noodles showed that the cooking quality and tensile properties of rice noodles were significantly enhanced as a result of the formation of double networks between the single helices produced by the high-temperature dissociation of CD and the starch in rice flour [13]. However, no work has been done to compare the effect of CD on the microstructure and quality attributes of rice noodles before and after thermal sterilization.

Herein, rice noodles without and with sterilization, with different CD contents from 0% to 2.0%, were studied. Our assumption was that CD, a polyhydroxy thermally set gel polysaccharide, can interact intensely with rice starch in rice noodles by hydrogen bonding, thereby forming a denser gel structure and improving their quality characteristics. In order to verify our hypothesis, we conducted a comprehensive investigation into how CD and thermal treatment influence the structure and rheological, textural, and cooking properties of rice noodle systems. This study provides a thorough and in-depth exploration aimed at enhancing the quality attributes of fresh rice noodle products, providing insights into innovative approaches for rice-based food across the industry field.

## 2. Materials and Methods

### 2.1. Materials

Rice was obtained from Guilin Dingyin Food Co., Ltd. (Guilin, China). Curdlan (87.6% purity, *M*_w_ = 9.14 × 102 Da) was sourced from Shandong Qidi Food Technology Co., Ltd. (Heze, China). Other reagents utilized in this research were of analytical grade.

### 2.2. Preparation of Samples

For rheological experiments, the slurries of rice flour (40 wt%) mixed with different proportions (0%, 0.1%, 0.5%, 1.0%, and 2.0%) of CD were prepared. Firstly, the CD was dispersed in water at 25 °C with stirring for 30 min, followed by blending with rice flour and stirring for an additional 30 min at the same temperature.

Different rice noodles with the same CD content and total concentration were prepared as follows: First, 8% of rice flour was blended with water at 45 °C in a 1:5 (*w/w*) ratio and stirred constantly for 1 min at 95 °C to create a pre-gelatinized rice flour paste. Afterward, the paste was blended with CD, the remaining rice flour, and water at room temperature. The mixed slurry was transferred into a mold that held 10 strips sized 20 cm × 1 cm, followed by steaming for 12 min at 100 °C, cooling for 10 min at room temperature, and, subsequently, covering it with plastic film and chilling it at 4 °C for 5 h, ultimately yielding homogeneous rice noodles. The samples were treated at 95 °C for 30 min to obtain sterilized rice noodles.

### 2.3. Rheological Test

Rheological assessments were carried out using a strain-controlled rheometer (MCR302, Anton Paar, Graz, Austria) equipped with parallel plates featuring a 1 mm gap and a 50 mm diameter. The mixed slurry was carefully placed between the two plates, and a small amount of silicone oil was used around the edges to prevent moisture evaporation. Prior to taking measurements, the samples were initially heated to 95 °C at a rate of 10 °C/min, held at this temperature for 5 min, and subsequently cooled down to 25 °C at the same rate, allowing for CD gelation.

Frequency sweep experiments were conducted from 0.1 rad/s to 100 rad/s under 1.0% strain at 25 °C. The power-law formulas were applied to characterize the frequency dependence of storage and loss moduli (*G*′ and *G*″). Equations (1) and (2) represent the relationships between log *G*′ and log *ω* and log *G*″ and log ω, from which the slopes (*n*′ and *n*″) and intercepts (*G*_0_′ and *G*_0_″) can be determined.

(1) $$G^{\prime }=G_{0}^{\prime }\omega^{n^{\prime }}$$

(2) $$G^{\prime \prime }=G_{0}^{\prime \prime }\omega^{n^{\prime \prime }}$$

### 2.4. Kinetic Analysis

In order to analyze the gel generation procedure of rice noodles, a constant-speed cooling method was employed, known for its effectiveness in observing gel formation dynamics. Samples were dropped from 95 °C to 25 °C at a cooling speed of 2 °C/min under constant conditions of 1.0% strain and 1 Hz frequency. Silicone oil was used around the edges of the sample to avoid water evaporation. Average structure developing rate (*SDR*_a_) and instant structure developing rate (*v*_g_) were used to evaluate the changes in storage modulus during gel formation. These two parameters can be calculated using Equations (3) and (4) below:

(3) $${SDR}_{a}=\frac{{G^{\prime }}_{end}-{G^{\prime }}_{ist}}{t_{end}-t_{ist}}$$

(4) $$v_{g}=\frac{dG^{\prime }}{dt}$$

*G*′*_ist_* and *G*′*_end_* denote the storage modulus (Pa) at the starting and ending temperatures, and *t*_ist_ and *t_end_* denote the time (s) at the starting and ending temperatures.

### 2.5. Scanning Electron Microscopy (SEM)

Freeze-dried rice noodle samples were first coated with gold. Subsequently, the fracture surfaces of the rice noodles were observed at 500× magnification employing a JSM-7500F scanning electron microscope instrument (Japan Electronic Instruments Co. Ltd., Akishima, Tokyo, Japan) operated at a 5 kV testing voltage.

### 2.6. Small-Angle X-Ray Scattering (SAXS)

SAXS analysis was performed with a Nano-inXider device (Xenocs, Grenoble, France) to study the microstructure of rice noodles. The water content of freeze-dehydrated rice noodles was regulated to 70% and allowed to keep for 12 h at room temperature. A suitable quantity of the sample was subsequently positioned on the sample holder and underwent testing for a duration of 5 min per trial, with air serving as the background. The collection of SAXS results was conducted at least thrice, covering a q range spanning from 0.007 Å^−1^ to 0.370 Å^−1^. All data were background-subtracted for the following analysis.

Using Equation (5), the Porod slope α is calculated, which can be further used to determine fractal dimension (D):

(5) $$I\left(q\right)\propto q^{-\alpha }$$

In this equation, *I* represents the intensity of the SAXS signal, *q* denotes the scattering vector, *α* is an exponent that corresponds to the Porod slope, and *d* is calculated using the equation *d* = 2π/*q*, which represents the average thickness of crystalline lamellae.

### 2.7. Fourier-Transform Infrared (FTIR)

FTIR data of freeze-dried rice noodles were obtained employing a Nicolet iS 10 spectrometer (Thermo, Waltham, MA, America) equipped with an ATR attachment. The scan parameters were configured to perform 64 scans, covering the range of 600–4000 cm^−1^ with a resolution of 4 cm^−1^. Baseline correction was applied to the whole spectrum using OMNIC 8.0 software. Every sample was measured at least thrice to ensure accuracy and reliability.

### 2.8. Textural Analysis

For textural tests, rice noodle samples were kept for 3 min in boiled water, followed by immersion for 1 min in cold water and gentle drying to remove excess water from the surface. Textural measurements were conducted employing a TA.XT PlusC textural apparatus (Stable Micro System Ltd., Surrey, UK) in TPA analysis pattern. Three sample strips, each measuring 25 mm × 10 mm and having the same thickness, were positioned side-by-side on the sample stage. These strips underwent two rounds of compression using a P/36R probe, with a trigger force set at 5 g. The measurement velocity was set to 1 mm/s. The deformation extent was defined as 30% of the sample height. Each sample was measured at least 5 times, with each measurement having a 5 min evaluation period.

### 2.9. Cooking Characteristics

The cooking quality of rice noodle samples was assessed following a previous technique (Kim et al., 2014) with slight alterations [14]. Ten rice noodle strips (*m*_0_) were boiled for 5 min in 500 mL water. Subsequently, the rice noodles were removed, drained to remove surface water, and then weighed to determine their mass (*m*_1_). The cooking water was poured into a 500 mL volumetric flask, shaken, and a 50 mL suspension was transferred to a dry, clean beaker (*m*_2_), then placed in a drying oven at 105 °C until a stable weight (*m*_3_) was reached. The water absorption rate (WAR) of the samples can be determined by Equation (6), and their cooking loss rate (CLR) can be calculated using Equation (7):

(6) $$WAR\left(\%\right)=\frac{m_{1}-m_{0}}{m_{0}}\times 100\%$$

(7) $$CLR\left(\%\right)=\frac{m_{3}-m_{2}}{m_{0}}\times 100\%$$

Ten intact rice noodles were boiled for 2 min in water (the noodle/water mass ratio was 15), after which they were removed and drained of surface water. The water used for cooking was gathered, of which the turbidity was measured by determining the absorbance at 675 nm using a UV–vis spectrophotometer (UV-5100B, Shanghai Metash Instruments Co., Ltd., Shanghai, China). Equation (8) was used to calculate the cooking breakage rate (CBR) of rice noodles:

(8) $$CBR\left(\%\right)=\frac{m_{4}}{m_{4}+m_{5}}\times 100\%$$

where *m*_4_ and *m*_5_ represent the weights of rice noodle strips less than 10 cm and rice noodle strips no less than 10 cm after boiling, respectively.

### 2.10. Sensory Evaluation

For each sample, 30 noodle strips were prepared by cooking and then cooling for 3 min. Sensory evaluation was conducted at the food laboratory in the Institute of Food Science and Technology, Chinese Academy of Agricultural Sciences, Beijing, China, using a modified method [15]. The evaluation panel, consisting of 15 males and 15 females (including students and teachers), completed the assessment within 10 min. The evaluated parameters included appearance (color and structural integrity), odor, taste, and palatability (softness and hardness). A 100-point scale was used for scoring, with the following point allocations: appearance (0–30), odor (0–20), taste (0–20), and palatability (0–30) [15]. Detailed sensory scoring criteria for the samples are provided in Table S1.

### 2.11. Statistical Analysis

All measurements were performed at least three times, and the data were analyzed using SPSS 16.0 software. The results are presented as mean ± standard deviation. Mean comparisons were conducted using one-way ANOVA followed by Duncan’s test, with a 95% confidence interval. A *p*-value of less than 0.05 was considered statistically significant.

## 3. Results and Discussion

### 3.1. Rheological Study of Rice Flour Gels Containing Curdlan

**Figure 1:**
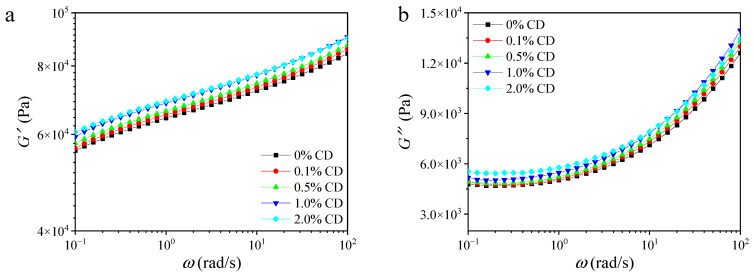
Storage modulus (*G*′, denoted by squares) and loss modulus (*G*″, denoted by circles) as a function of frequency (*ω*) for rice flour gels with different proportions (0%, 0.1%, 0.5%, 1.0%, and 2.0%) of CD (**a**,**b**). The data was obtained from a frequency sweep analysis of the *G*′ and *G*″ of the sample structure at different frequencies.

Figure 1 exhibits the *G*′ and *G*″ values of rice flour gels containing different CD contents as a function of frequency, which are used to characterize the energy that can be stored in the elastic structure of the material (reflecting elasticity or stiffness) and the energy that will be dissipated within the material by means of heat due to viscous flow [16,17]. Throughout the frequency range tested, *G*′ exceeded *G*″ for all rice flour gel samples with different CD proportions, indicating typical solid-like behavior [18]. The moduli (*G*′ and *G*″) of gel samples increased with incremental CD content, indicating that CD effectively enhanced the gel strength and solid-like behavior of rice flour gels.

Table 1 presents the *n*′, *G*_0_′, *n*″, and *G*_0_″ values for gel samples at 25 °C, derived over a frequency range spanning from 10^-1^ to 10^2^ rad/s. The *n*″ values were notably higher than *n*′ for all samples, indicating a stronger frequency dependence of *G*″ compared to *G*′, highlighting the predominant elasticity of the samples over viscosity [19]. In addition, *G*_0_″ values were significantly lower than *G*_0_′, confirming the solid-like behavior of these gel samples [20]. A higher CD content resulted in higher *G*_0_′ values and lower *n*′ values, indicating that CD enhanced the solid-like behavior and reduced the frequency dependence of the pure rice flour gel for *G*′.

### 3.2. Kinetic Analysis of Gel Generation

**Figure 2:**
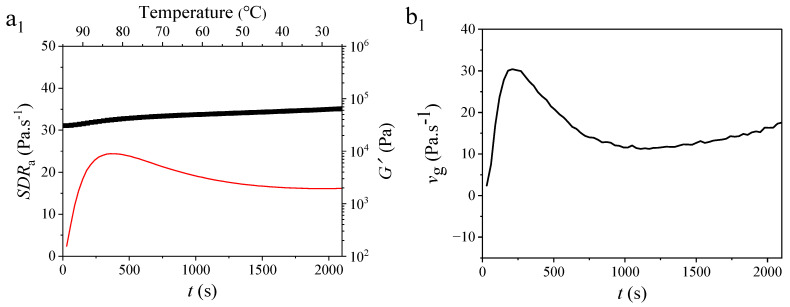

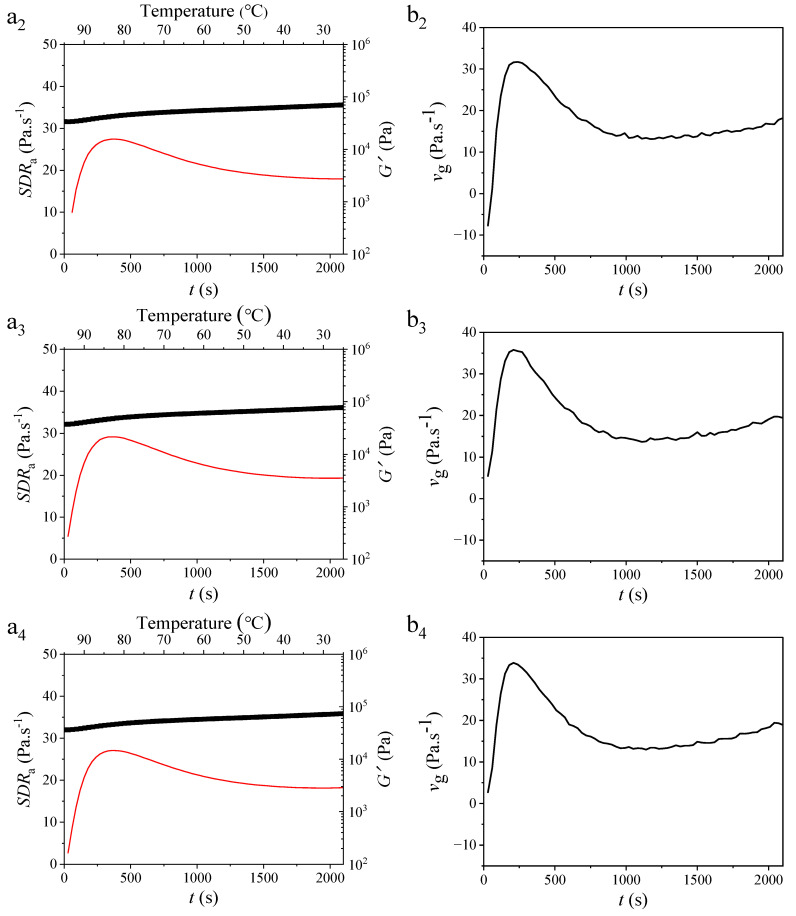

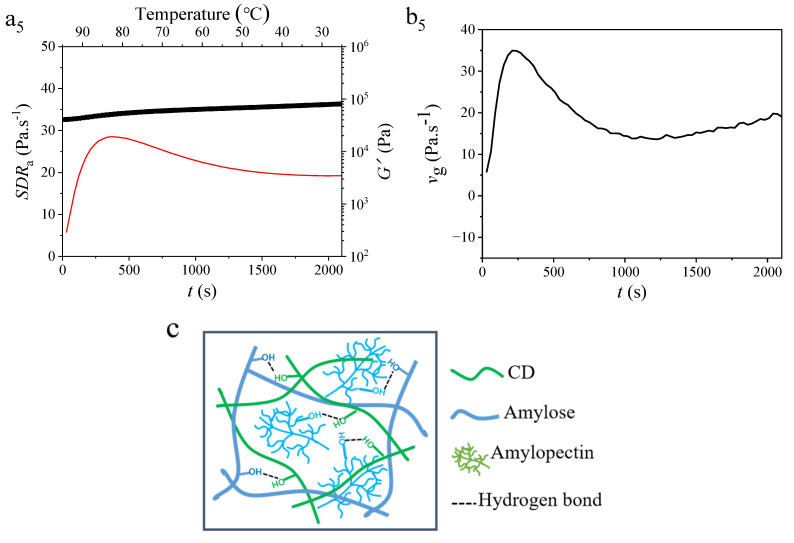
Temperature- and time-dependent curves of *SDR*_a_ (average structure developing rate denoted by red) and *G*′ (storage modulus denoted by black) (**a_1_**–**a_5_**) and time-dependent *v*_g_ (instant structure developing rate) curves (**b_1_**–**b_5_**) for rice flour gels incorporating different proportions (0%, 0.1%, 0.5%, 1.0%, and 2.0%) of CD. Structural schematic representation of rice flour gel with CD (**c**). The analysis results of gel formation kinetics characterize the formation process of rice flour gel from multiple perspectives.

Figure 2 shows the *SDR*_a_, *G*′, and *v_g_* curves for rice flour gels with different CD contents. *v*_g_ and *SDR*_a_ characterize the relative gel formation rate of gel samples at specific time points over time [21,22]. The G′ of the pure rice flour gel sample continuously increased with the temperature dropped, in accordance with both *SDR*_a_ and *v*_g_ values being greater than 0. This observation suggests that rice starch predominantly acted as a cooling-set gel. The *SDR*_a_ and *v_g_* curves exhibited similar trends, with *G*′ first increasing and then decreasing as the temperature decreased. The growth rate of *G*′ peaked at 86.81 °C in the *v*_g_ curve, whereas the *SDR*_a_ curve showed a hysteresis phenomenon, reaching its peak at 82.8 °C.

Contrasted with the pure rice flour gel, the *SDR*_a_, *G*′, and *v*_g_ curves of the composite gels displayed an analogical trend as the temperature dropped, suggesting the dominant role of rice starch in the formation of composite gels. Interestingly, as the CD content increased, the peak temperature of the *v*_g_ curve remained relatively constant (86.81 °C, 86.82 °C, 87.82 °C, 87.82 °C, 87.82 °C, and 87.82 °C for rice flour gels with CD contents of 0%, 0.1%, 0.5%, 1.0%, and 2.0%, respectively). However, the *v*_g_ values (31.73, 35.8, 33.87, and 34.93) at the curve peak temperature of the composite samples were significantly higher than that of the pure rice flour gel (30.4). This suggests that the inclusion of CD enhanced rice starch gel formation in rice flour gels. This enhancement can be attributed to CD exposing more hydroxyl groups during gel formation through hydrophobic interaction, thereby promoting the generation of intermolecular hydrogen bonds during cooling (Figure 2c). The occurrence of this phenomenon may also be attributed to entanglement among the single helices dissociated from the triple helices of CD at high temperatures, starch chains, and rice protein chains. The entanglement of molecular chains is enhanced with increasing CD content. The chain entanglement effect is evident in the early stages of gel formation, facilitating rapid establishment of a three-dimensional gel network structure.

### 3.3. Structures and Application-Related Attributes of Rice Noodles

#### 3.3.1. Supramolecular Structure

**Figure 3:**
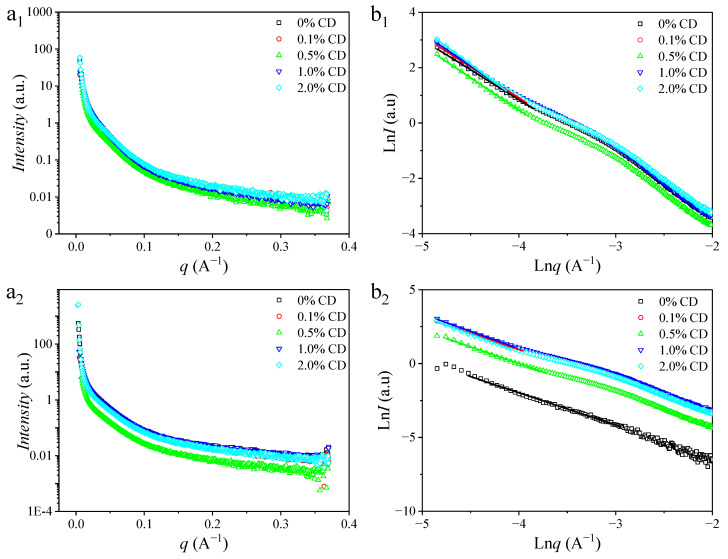
SAXS patterns for rice noodles containing different proportions (0%, 0.1%, 0.5%, 1.0%, and 2.0%) of CD without (**a_1_**,**a_2_**) and with sterilization (**b_1_**,**b_2_**). SAXS analyzes the fractal structure and lamellar ordered structure of rice noodles. *I* represents the intensity of the SAXS signal, and *q* denotes the scattering vector.

The SAXS curves for rice noodles containing different amounts of CD, both sterilized and unsterilized, are shown in Figure 3. Across the entire testing range, all samples exhibited similar trends where the scattering intensity decreased notably with increasing *q*. This phenomenon was primarily related to the generation of large-scale network structures or intermolecular aggregation [23]. Notably, a “shoulder-like” scattering peak appeared in the *q* scope of 0.03–0.06 Å^−1^ for all samples.

For rice noodles without and with sterilization, the location of the “shoulder-like” band shifted towards high *q* values with incremental CD content. This shift suggests a diminishment in the dimension of microcrystalline regions in rice noodles, as indicated by the average thickness (*d*) and position (*q*) of the lamellar order structure listed in Table 2. This change can be attributed to two main factors. Firstly, the generation of intermolecular hydrogen bonds between CD and amylopectin blocks the generation of amylose intermolecular hydrogen bonds, leading to a thinner crystal layer structure [1]. Secondly, minor components in rice noodles, such as rice protein, may increase steric hindrance between starch molecules, thereby impeding the formation of order structure. Comparatively, sterilized samples showed a decrease in the *d* value compared to unsterilized samples with equal CD content. This reduction could be on account of partial damage to hydrogen bonds formed in the gel process of rice noodles during thermal treatment resulting in a decrease of the ordered structure of sterilized rice noodles.

Fractal dimension (*D*) serves to characterize the density of gel network structures [24]. As shown in Table 2, the fractal dimension values of all samples are less than 3, indicating that these structures are mass fractals [25]. For both sterilized and non-sterilized rice noodles, the *D* value increased with higher CD content, indicating the generation of a more compact network structure. This confirms that CD had a protective impact on the structural integrity of rice noodles. Comparing rice noodles with equal CD content, the *D* value of sterilized rice noodles is lower than that of non-sterilized rice noodles. This difference may be attributed to the thermal damage to hydrogen bonds in rice noodles during the heating process resulting in a reduction in the density of rice noodle gels with sterilization.

#### 3.3.2. Textural Properties

**Figure 4:**
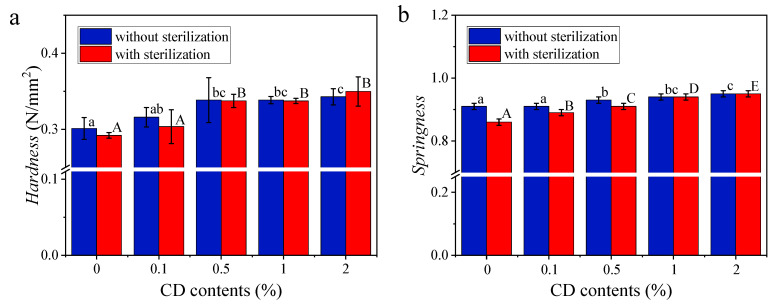

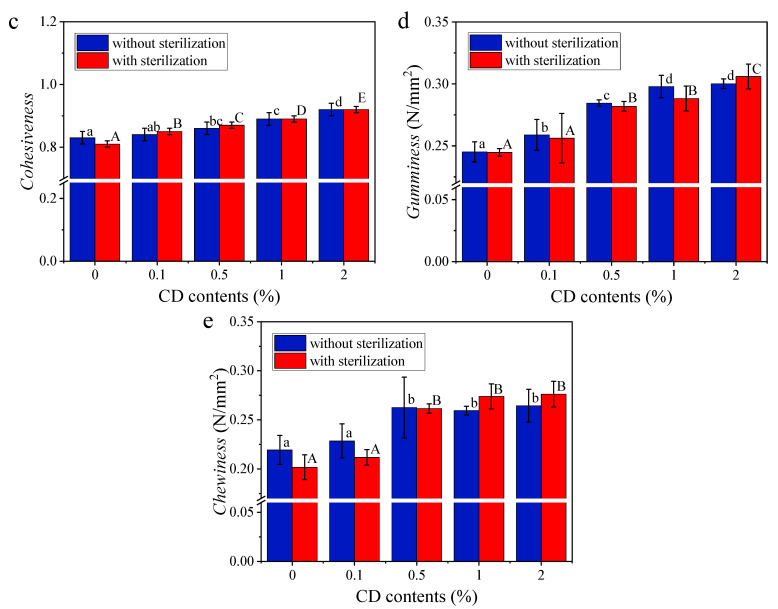
Textural characteristics (hardness, springiness, cohesiveness, gumminess, and chewiness) of unsterilized and sterilized rice noodles incorporating different proportions of CD (0%, 0.1%, 0.5%, 1.0%, and 2.0%) (**a**–**e**). The texture tests evaluate the hardness, springiness, cohesiveness, gumminess, and chewiness of rice noodles under the TPA mode. Significant differences (*p* < 0.05) of textural data for rice noodle samples without and with sterilization are respectively indicated by different lowercase letters (a–d) or by using different uppercase letters (A–E).

Figure 4 depicts the textural attributes of rice noodles integrating with proportions of CD, both without and with sterilization. The parameters measured, including cohesiveness, springiness, hardness, chewiness, and gumminess, all exhibit an upward trend with increasing CD content, irrespective of sterilization. As shown by the FTIR results, the incorporation of CD enhanced intramolecular and intermolecular hydrogen bonding in rice noodles, resulting in the forming of a more elastic and stronger gel structure, which could account for the increasing textural parameters observed here.

In samples without sterilization, the hardness, springiness, and cohesiveness were higher compared to sterilized samples with an equal CD content (CD content ≤ 1.0%), particularly evident in pure rice noodles. This difference indicates that the thermal process damaged the structural integrity of rice noodles, resulting in reduced gel strength. Additionally, the gumminess of samples without sterilization with a CD content ≤ 1.0% was also higher than sterilized samples, indicating that thermally treated rice noodles exhibited a softer and more fragile quality [3]. Nevertheless, when the CD content was raised to 2.0%, the hardness, gumminess, and chewiness of unsterilized samples were lower than those of sterilized samples, without significant differences observed in springiness and cohesiveness between the two. This suggests that the addition of CD can mitigate or even reverse the worsening quality of rice noodles caused by heating treatment.

#### 3.3.3. FTIR

**Figure 5:**
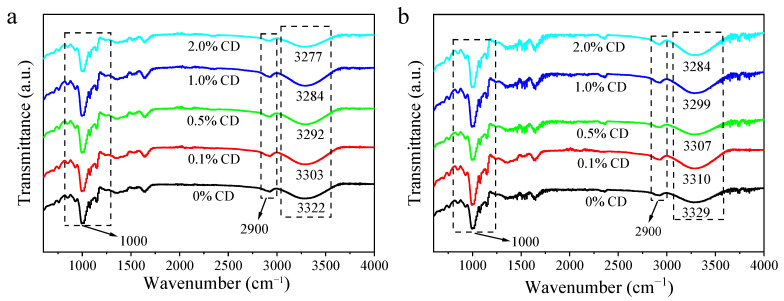
FTIR curves of unsterilized (**a**) and sterilized (**b**) rice noodles containing different proportions (0%, 0.1%, 0.5%, 1.0%, and 2.0%) of CD. The changes of hydrogen bond characteristic peaks of different rice noodle samples were analyzed by FTIR results.

Figure 5 displays the FTIR curves for rice noodles with different CD contents, both without and with sterilization. It is evident that all samples presented an analogous trend without the appearance of new absorption peaks. This observation indicates that there were no obvious covalent bond formations or chemical structure alterations in the process of rice noodle preparation.

In all samples, a prominent peak was evident at approximately 1000 cm^−1^, which is ascribable to the stretching oscillation of C—O—C, C—O, and β-glycosidic linkages of polysaccharide chains [26,27]. Additionally, the peak that appears at 2900 cm^−1^ can be linked to the stretching of the C—H (CH_2_) group [28]. Furthermore, a wide peak around 3300 cm^-1^ can be observed, corresponding to the presence of hydrogen bonds [29]. The shift of characteristic peaks related to hydrogen bonds towards lower wavenumbers is commonly used to indicate the formation of hydrogen bonds [30].

With incremental CD proportion, the peak characteristic of hydrogen bonds of rice noodles without and with sterilization shifted to lower wavenumbers. This shift suggests that CD effectively enhanced intramolecular and intermolecular hydrogen bonds in rice noodles [31]. Among rice noodles with equal CD content, the hydrogen-bond peak of sterilized samples shifted towards higher wavenumbers compared to samples without sterilization. This shift is most likely attributed to the thermal disruption of hydrogen bonds within rice noodles as a result of the heating process. This result is in agreement with the results from SAXS tests, indicating structural damage and quality deterioration in rice noodles after thermal treatment.

#### 3.3.4. SEM

**Figure 6:**
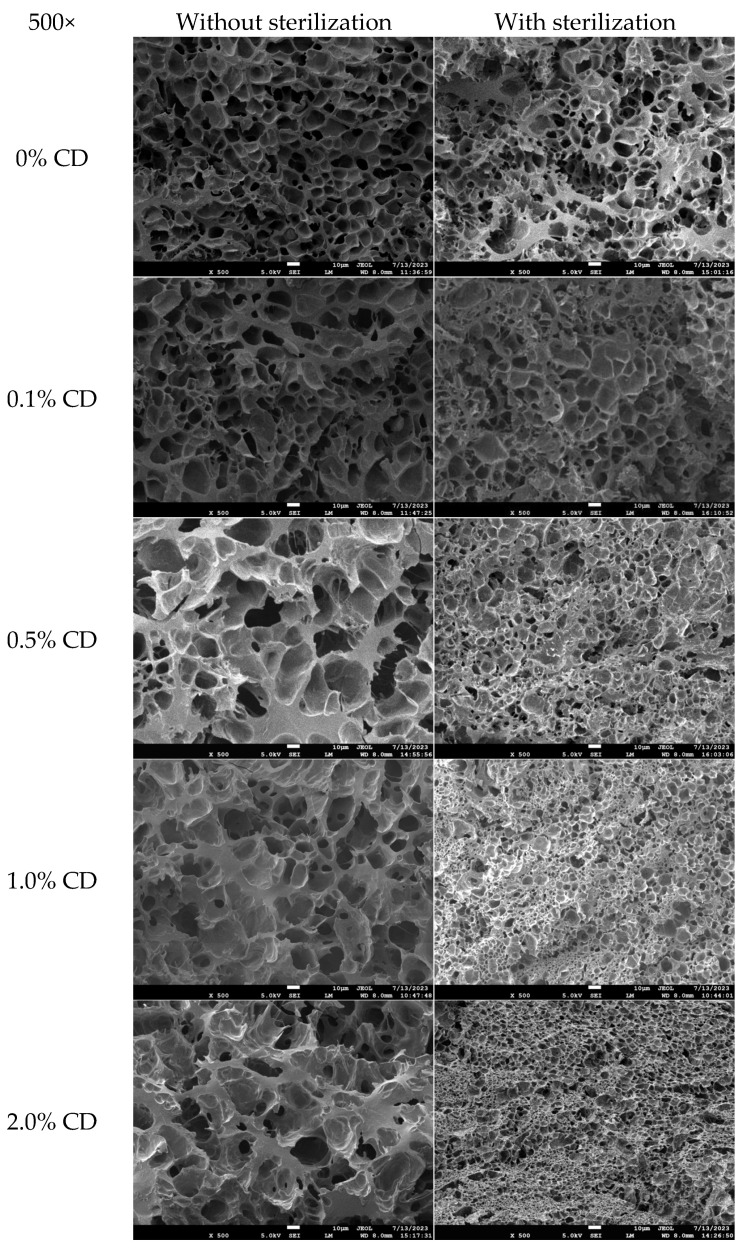
SEM images of unsterilized and sterilized rice noodles incorporating different proportions (0%, 0.1%, 0.5%, 1.0%, and 2.0%) of CD. The cross-section morphology of rice noodles gel samples at 500× was observed by SEM.

Figure 6 displays the SEM images of the cross-sectional areas of the rice noodles with different CD proportions, both without and with sterilization, as viewed under SEM. A beehive-like structure was shown in the SEM images of all samples. For rice noodles without sterilization, an increase in CD content resulted in larger network voids and thicker walls, suggesting that CD interacted with rice flour by forming inter-chain hydrogen bonds, as supported by FTIR. Interestingly, with increasing CD content, sterilized samples exhibited more uniform and denser voids. This indicates that CD, with its ability to form thermally set gels, significantly enhanced gel strength during the heating process.

For pure rice noodles, contrasted with unsterilized samples, sterilized samples suffered a secondary heating process, leading to lower structural integrity, likely resulting from the thermal spoilage of hydrogen bonds in rice noodles. However, an interesting phenomenon was observed in rice noodle samples containing the same CD content, both without and with sterilization. Samples with sterilization exhibited smaller and more uniform void sizes compared to samples without sterilization, particularly noticeable in samples with ≥ 0.5% CD content. This phenomenon can be related to the thermal gelation characteristic of CD, which favors the formation of a denser gel structure resulting from the secondary heating process. Previous studies primarily focused on how varying amounts of CD influence the structure and edible quality of sterilized fresh rice noodles [13]. However, there has been limited investigation into the impact of CD on the structure of rice noodles before and after undergoing a secondary heating process. In contrast, this current work provides a new strategy for the application of CD in starch-based foods requiring multiple heating (for gelatinization and sterilization).

#### 3.3.5. Cooking Characteristics

Table 3 presents the cooking characteristics of rice noodles integrating different proportions of CD, both without and with sterilization. Key parameters, including WAR, CLR, CBR, and turbidity, were assessed to evaluate the cooking characteristics of rice products. In both sterilized and non-sterilized samples, the WAR rose with higher CD content. This phenomenon can be attributed to CD’s hydrophilic characteristic, which facilitates the permeation of water molecules and enhances the water-holding capacity of rice noodles. Concurrently, the CLR, CBR, and turbidity gradually decreased with increasing CD content. This effect can be attributed to hydrogen bonding between the chains of rice starch (the primary ingredient in rice noodles) and CD, which strengthened the gel network structure and reduced the likelihood of rice noodle fracture during cooking. This trend is similar to previous research [13].

Compared to pure rice noodle samples without sterilization, sterilization increased the water absorption rate. The thermal process disrupted hydrogen bonds, releasing more hydroxyl groups that can combine with water molecules, thereby increasing water absorption. Conversely, the higher values of turbidity, CLR, and CBR recorded post-sterilization may be a result of the gel structure of rice noodles breaking down during the heat treatment process. These results are consistent with the results from textural tests.

However, with increasing CD content, differences in turbidity, CLR, and CBR between sterilized samples and pure rice noodles without sterilization were gradually reduced or even reversed, which has not been observed before. This indicates that adding enough CD can not only resist the damage of thermal sterilization to rice noodles but also enhance the cooking characteristics of pure rice noodles without sterilization. It is noteworthy that rice noodles with 2.0% CD content showed significant improvements in cooking properties due to enhanced gel network strength, as proved by the SAXS and FTIR tests.

#### 3.3.6. Sensory Quality

**Figure 7:**
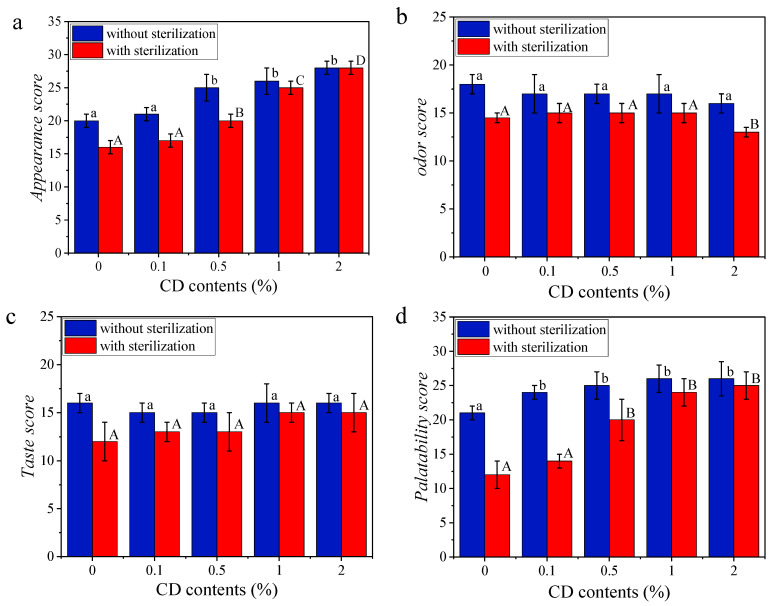
Sensory evaluation scores (appearance score, odor score, taste score, and palatability score) of unsterilized and sterilized rice noodles incorporating different proportions (0%, 0.1%, 0.5%, 1.0%, and 2.0%) of CD (**a**–**d**). The sensory quality of rice noodles gel samples can be quantified by sensory evaluation tests. Significant differences (*p* < 0.05) of sensory evaluation scores for rice noodle samples without and with sterilization are respectively indicated by different lowercase letters (a,b) or by using different uppercase letters (A–D).

Figure 7 shows the sensory qualities of rice noodles integrating different proportions of CD, both without and with sterilization. The appearance and palatability of sterilized and unsterilized rice noodles are better with the addition of CD. This may be due to the thermally irreversible gel property of CD, which plays an important role in protecting the chain network in rice noodles. Meanwhile, the differences between unsterilized and sterilized rice noodles in appearance and palatability gradually decreased with increasing CD content. However, for sterilized and unsterilized rice noodles, with the addition of CD, the taste had no significant difference. Compared with pure rice noodles without sterilization, the odor and taste of sterilized samples decreased significantly, indicating that the heat sterilization treatment had a destructive effect on the odor and taste of rice noodles. It is worth noting that increasing the content of CD to 2% significantly reduced the odor of sterilized rice noodles. Consequently, in industrial production, the addition of CD should not exceed 2%, as higher amounts may negatively impact consumer acceptance of rice noodle products. Based on an evaluation of four key indicators, the optimal amount of CD to be added is 1%, which aligns closely with the findings of a previous study that reported a similar value of 1.13% [13].

## 4. Conclusions

This work examines the impact of incorporating CD and subjecting rice noodles to thermal sterilization on their microstructure, chain interactions, and product quality. FTIR analysis demonstrated the fact that the incorporation of CD enhanced hydrogen bonding within the rice noodle matrix, leading to a denser gel structure, as confirmed by SAXS and SEM results. This structural enhancement correlated with improved textural and cooking characteristics of the rice noodles. Specifically, higher CD content resulted in lower frequency dependence, higher modulus, and solid-like behavior. Meanwhile, the incorporation of CD accelerated the gel structural formation of rice noodle gels.

Rice starch, the main component of rice noodles, naturally forms a gel that sets upon cooling. However, thermal treatment can disrupt these hydrogen bonds, damaging the gel’s structural integrity and leading to a fragile and soft texture with lower mechanical and cooking properties. Taking all factors into consideration, the incorporation of 1.0% CD mitigated these negative effects of thermal treatment. It significantly improved the microstructure and quality attributes of rice noodles, enhancing texture, cooking, and sensory characteristics. These findings offer ponderable viewpoints for enhancing the performance of rice-based food products and other cooling–setting gel systems. They underscore the potential of CD as a functional additive in food processing to optimize product quality under thermal conditions.

## Figures and Tables

**Table 1:** *n*′, *n*″, *G*_0_′, and *G*_0_″ for rice flour gels with different proportions (0%, 0.1%, 0.5%, 1.0%, and 2.0%) of CD at 25 °C as determined from Equations (1) and (2) within the frequency interval from 10^−1^ to 10^2^ rad/s.

CD Content	*n*′	*G*_0_′ (Pa)	*R* ^2^	*n*″	*G*_0_″ (Pa)	*R* ^2^
0%	0.055 ± 0.000 ^a^	65,991 ± 1670 ^a^	0.9956	0.148 ± 0.003 ^a^	5603 ± 140 ^a^	0.9127
0.1%	0.056 ± 0.000 ^a^	65,761 ± 1065 ^a^	0.9950	0.152 ± 0.004 ^a,b^	5639 ± 74 ^a^	0.9173
0.5%	0.055 ± 0.001 ^a^	66,163 ± 269 ^b^	0.9953	0.147 ± 0.003 ^b,c^	5576 ± 102 ^b,c^	0.9105
1.0%	0.055 ± 0.002 ^a^	66,895 ± 1995 ^b,c^	0.9957	0.142 ± 0.009 ^c,d^	5702 ± 334 ^a,b^	0.9086
2.0%	0.058 ± 0.001 ^a,b^	68,500 ± 417 ^d^	0.9969	0.135 ± 0.002 ^d^	6400 ± 444 ^c^	0.9106

Note: Results are displayed as mean ± SD. Significant differences (*p* < 0.05) are indicated by contrasting lowercase letters within a single column.

**Table 2:** Supramolecular structure parameters of unsterilized and sterilized rice noodles incorporating different contents of CD (0%, 0.1%, 0.5%, 1.0%, and 2.0%). *D*, *q*, and *d*, respectively, are the fractal dimension, position, and average thickness of the lamellar order structure.

CD Content	Sterilization	D	q (Å–1)	d (nm)
0%	without	2.19 ± 0.01 ^a^	0.0563 ± 0.0004 ^a^	11.16 ± 0.08 ^a^
	with	2.17 ± 0.01 ^A^	0.0595 ± 0.0005 ^A^	10.55 ± 0.09 ^A^
0.1%	without	2.23 ± 0.00 ^b^	0.0579 ± 0.0002 ^b^	10.85 ± 0.04 ^b^
	with	2.22 ± 0.01 ^B^	0.0609 ± 0.0007 ^B^	10.32 ± 0.12 ^B^
0.5%	without	2.33 ± 0.00 ^c^	0.0588 ± 0.0006 ^c^	10.67 ± 0.11 ^c^
	with	2.26 ± 0.00 ^C^	0.0630 ± 0.0003 ^C^	9.97 ± 0.05 ^C^
1.0%	without	2.37 ± 0.01 ^d^	0.0596 ± 0.0003 ^c^	10.53 ± 0.04 ^c^
	with	2.33 ± 0.01 ^D^	0.0641 ± 0.0003 ^D^	9.80 ± 0.05 ^D^
2.0%	without	2.58 ± 0.01 ^e^	0.0609 ± 0.0006 ^d^	10.32 ± 0.10 ^d^
	with	2.42 ± 0.01 ^E^	0.0647 ± 0.0005 ^D^	9.70 ± 0.07 ^D^

Note: Results are displayed as mean ± SD. Significant differences (*p* < 0.05) of SAXS data for rice noodle samples without and with sterilization are indicated by contrasting lowercase letters (a–e) or by using different uppercase letters (A–E) within a single column.

**Table 3:** Cooking characteristics (including water absorption rate (WAR), cooking loss rate (CLR), cooking breakage rate (CBR), and turbidity) of unsterilized and sterilized rice noodles incorporating different proportions (0%, 0.1%, 0.5%, 1.0%, and 2.0%) of CD.

CD Content	Sterilization	WAR (%)	CLR (%)	CBR (%)	Turbidity (Au)
0%	without	30.87 ± 1.53 ^a^	0.35 ± 0.05 ^a^	35.71 ± 4.62 ^a^	0.356 ± 0.038 ^a^
	with	35.53 ± 4.24 ^A^	0.66 ± 0.05 ^A^	60.97 ± 1.68 ^A^	0.624 ± 0.067 ^A^
0.1%	without	31.04 ± 4.52 ^a^	0.35 ± 0.05 ^a^	31.07 ± 2.95 ^a^	0.373 ± 0.021 ^a^
	with	36.01 ± 2.31 ^A^	0.54 ± 0.06 ^B^	55.60 ± 5.34 ^A^	0.532 ± 0.058 ^B^
0.5%	without	31.10 ± 2.21 ^a^	0.34 ± 0.05 ^a^	30.30 ± 4.20 ^a,b^	0.363 ± 0.127 ^a^
	with	37.34 ± 2.72 ^A^	0.29 ± 0.03 ^C^	37.60 ± 7.23 ^B^	0.487 ± 0.018 ^B^
1.0%	without	31.13 ± 1.92 ^a^	0.28 ± 0.04 ^a,b^	29.32 ± 2.90 ^a,b^	0.223 ± 0.017 ^b^
	with	39.33 ± 1.63 ^A^	0.27 ± 0.02 ^C^	34.30 ± 2.96 ^B^	0.389 ± 0.037 ^C^
2.0%	without	36.44 ± 0.98 ^b^	0.23 ± 0.00 ^b^	23.50 ± 3.36 ^b^	0.218 ± 0.015 ^b^
	with	40.31 ± 2.84 ^A^	0.21 ± 0.01 ^C^	15.42 ± 4.54 ^C^	0.217 ± 0.008 ^C^

Note: Results are displayed as mean ± SD. Significant differences (*p* < 0.05) of cooking data for rice noodle samples without and with sterilization are respectively indicated by contrasting lowercase letters (a,b) or by using different uppercase letters (A–C) within a single column.

## Data Availability

The original contributions presented in this study are included in the article/Supplementary Material. Further inquiries can be directed to the corresponding authors.

## Supplementary Materials

The following supporting information can be downloaded at: https://www.mdpi.com/article/10.3390/foods14040674/s1, Table S1. Sensory scoring rules for rice noodles.
