# Supplementary material for: Curdlan Incorporation Enhances the Cooking, Rheological, and Textural Attributes of Thermally Sterilized Rice Noodles
Source: Foods. 2025 Feb 17;14(4):674. doi: 10.3390/foods14040674 (PMC11854599; doi:10.3390/foods14040674)
Supplement: Supplementary file 1 [file foods-14-00674-s001.zip › foods-3417985-supplementary.pdf]

- Supplementary material -

Curdlan Incorporation Enhances the Cooking, Rheological, and Textural Attributes of Thermally Sterilized Rice Noodles

Jing Wang [1,2], Yongxin Liu [2], Qingjie Sun [2], Man Li [2,*], Yanfei Wang [1,2,*] and Fengwei Xie [3]

[1] Ministry of Agriculture and Rural Affairs Key Laboratory of Agro-Products Processing, Institute of Food Science and Technology, Chinese Academy of Agricultural Sciences, Beijing 100193, China
[2] College of Food Science and Engineering, Qingdao Agricultural University, Qingdao 266109, China
[3] Nottingham Ningbo China Beacons of Excellence Research and Innovation Institute, University of Nottingham Ningbo China, 211 Xingguang Road, Ningbo 315048, China
* Correspondence: manli@qau.edu.cn (M.L.); yanfeiwang@qau.edu.cn (Y.W.)

List of supplementary tables

Table S1. Sensory scoring rules for rice noodles.

TABLE

Table S1. Sensory scoring rules for rice noodles.

**Index (score)**	**Description**
Appearance (30)	color (10): Off-white (8-10), Normal (4-7), Heterochromatic color (0-3)<br>structural integrity (20): epidermis without cracking and not easily broken (11-20), A small amount of cracking of the epidermis without breaks (6-10), epidermis with cracking and easily broken (0-5)
Odor (20)	rice aroma (16-20)<br>light rice flavor (11-15)<br>No rice fragrance, no peculiar smell (5-10)<br>No rice fragrance, peculiar smell (0-4)
Taste (20)	Stronger rice flavor when chewing (16-20)<br>Faint rice flavor when chewing (11-15)<br>No rice flavor, no peculiar smell when chewing (5-10)<br>No rice flavor but peculiar smell when chewing (0-4)
Palatability (30)	Moderate hardness and softness (20-30), Slightly soft or slightly hard (10-19), Very soft or very hard (0-9)
